# Sensitive and accurate DNA metabarcoding of parasitic helminth mock communities using the mitochondrial rRNA genes

**DOI:** 10.1038/s41598-022-14176-z

**Published:** 2022-06-15

**Authors:** Abigail Hui En Chan, Naowarat Saralamba, Sompob Saralamba, Jiraporn Ruangsittichai, Kittipong Chaisiri, Yanin Limpanont, Vachirapong Charoennitiwat, Urusa Thaenkham

**Affiliations:** 1 Department of Helminthology, Faculty of Tropical Medicine, Mahidol University, Bangkok, Thailand; 2 Department of Molecular Tropical Medicine and Genetics, Faculty of Tropical Medicine, Mahidol University, Bangkok, Thailand; 3 Mathematical and Economic Modelling (MAEMOD), Mahidol Oxford Tropical Medicine Research Unit, Faculty of Tropical Medicine, Mahidol University, Bangkok, Thailand; 4 Department of Medical Entomology, Faculty of Tropical Medicine, Mahidol University, Bangkok, Thailand; 5 Department of Social and Environmental Medicine, Faculty of Tropical Medicine, Mahidol University, Bangkok, Thailand

**Keywords:** Next-generation sequencing, Genetic markers

## Abstract

Next-generation sequencing technologies have accelerated the pace of helminth DNA metabarcoding research, enabling species detection in bulk community samples. However, finding suitable genetic markers with robust species-level resolution and primers targeting a broad species range among parasitic helminths are some of the challenges faced. This study aimed to demonstrate the potential use of the mitochondrial 12S and 16S rRNA genes for parasitic helminth (nematodes, trematodes, cestodes) DNA metabarcoding. To demonstrate the robustness of the 12S and 16S rRNA genes for DNA metabarcoding, we determined the proportion of species successfully recovered using mock helminth communities without environment matrix and mock helminth communities artificially spiked with environmental matrices. The environmental matrices are human fecal material, garden soil, tissue, and pond water. Our results revealed the robustness of the mitochondrial rRNA genes, through the high sensitivity of the 12S rRNA gene, and the effectiveness of the 12S and 16S primers targeting platyhelminths. With the mitochondrial rRNA genes, a broad range of parasitc helminths were successfully detected to the species level. The potential of the mitochondrial rRNA genes for helminth DNA metabarcoding was demonstrated, providing a valuable gateway for future helminth DNA metabarcoding applications like helminth detection and biodiversity studies.

## Introduction

The disparity between the projected estimate of helminth species and those described is huge, with approximately only 15% currently defined out of 100,000 projected helminth species^1^. Inhabiting a diverse range of habitats and hosts, parasitic helminths are well-known to cause diseases in humans, animals, and plants, and the secondary consequences of helminthic diseases can impede the development of various economic and industrial sectors globally^2–4^. Albeit the public health and economic importance of parasitic helminths, species identification through morphology remains the gold standard. Recently, the popularity of DNA metabarcoding has been gaining traction, with applications in many diverse fields of biology, including the detection of helminths in the environment^5–8^. The term DNA metabarcoding was first introduced by Teberlet et al. (2012) to designate high throughput multi-species identification^9^. Species identification can be performed from bulk samples of entire organisms or environment samples via next-generation sequencing (NGS) technologies. Though DNA metabarcoding, it (1) decreases the reliance on taxonomic expertise and ambiguous morphological characters, (2) allows for simultaneous detection of more than one species in a single reaction, thus saving cost, (3) increases the sensitivity for detection allowing trace amounts of the target organism’s DNA to be detected, and (4) allows for a non-invasive method for organism surveillance^10–13^.

The applications of helminth DNA metabarcoding are immense, including research on biodiversity and conservation of helminths in various habitats, biomonitoring of environments through helminth diversity, and the non-invasive detection and surveillance of parasitic helminths^7,14–16^. Although DNA metabarcoding holds vast potential and is a promising alternative for species identification, its application for parasitic helminths is still at an early stage. Various considerations like performing prior evaluation via mock communities, having a suitable helminth isolation and DNA extraction strategy, selecting a suitable genetic marker, sensitivity, and specificity of available primers, and type of bioinformatics analysis, are crucial for a successful DNA metabarcoding approach^17,18^.

Various genetic markers have been utilized for helminth DNA metabarcoding, and popular ones include the nuclear 18S ribosomal RNA (rRNA) genes, the nuclear internal transcribed spacer 2 (ITS2) region, and the mitochondrial Cytochrome *c* oxidase subunit I (*COI*) gene^7,19–21^. DNA metabarcoding of soil and marine nematodes has been performed using the nuclear 18S rRNA gene through primers specifically targeting soil and marine nematodes^7,19^. However, the use of the 18S rRNA gene as a DNA metabarcoding marker is limited by its taxonomic resolution due to the high sequence conservation of the genetic marker. Its specificity remains an issue as studies have revealed that only a small proportion of sequence reads are specific to nematodes^22,23^. In addition to environmental samples, the non-invasive sampling of gastrointestinal nematodes in animal hosts through fecal samples emphasizes the potential of helminth DNA metabarcoding^24,25^. Studies have investigated the impact of routine anti-parasitic treatment on parasitic nematode communities of cattle, illustrating the power of DNA metabarcoding in monitoring anti-parasitic resistance in livestock^26^. The detection of gastrointestinal nematodes of livestock using the ITS2 region, more commonly known as ‘nemabiome’ is also popular^21^. Although the species-level taxonomic resolution is robust using the ITS2 region, its high variability and varying lengths between diverse nematode taxa make it a challenging genetic marker to use. Similarly, the high sequence variability of the mitochondrial *COI* gene in helminths restricts its use as an optimal DNA metabarcoding marker despite having better species-level resolution than the nuclear 18S rRNA gene^27,28^. PCR amplification bias is prominent, selectively amplifying only some species, thus limiting the scope of species detection for DNA metabarcoding studies.

Utilizing the mitochondrial 12S and 16S rRNA genes, primers targeting parasitic nematodes and trematodes were recently developed^29,30^. The potential of using the mitochondrial rRNA genes for helminth molecular-based studies was illustrated by its sensitivity to detect various life-cycle stages of parasitic helminths with robust species-level taxonomic resolution and the ability to amplify DNA for a broad range of nematodes and trematodes. To date, most helminth DNA metabarcoding studies have focused on characterizing nematode communities in soil and marine environments with the nuclear 18S rRNA gene. Moreover, studies are few and far between for DNA metabarcoding of platyhelminths. Of late, Douchet et al. (2021) revealed the power of the mitochondrial 16S rRNA gene for environment DNA (eDNA) metabarcoding of parasitic trematode communities in freshwater systems, further substantiating the potential of the mitochondrial rRNA genes for parasitic helminth DNA metabarcoding^31^.

Here, we aim to demonstrate the potential use of the mitochondrial 12S and 16S rRNA genes for parasitic helminth (nematodes, trematodes, and cestodes) DNA metabarcoding. We determine the robustness of the 12S and 16S rRNA genes and primers for species identification using mock helminth communities in artificially spiked environment matrices. Using mock helminth communities presents a beneficial opportunity to evaluate the potential suitability of the mitochondrial rRNA genes for DNA metabarcoding. With known species in mock communities, it can aid to determine PCR amplification efficiency or detect possible bias against certain species, and also test the sensitivity and robustness of these new primers^32^. Moreover, as there is currently no available DNA metabarcoding strategy collectively targeting parasitic nematodes and platyhelminths together, we provide an avenue for, as well as alternative genetic markers for DNA metabarcoding of parasitic helminths.

## Results

### Data characteristics

From the 20 sets of mock communities for the two genetic markers (12S-platyhelminth, 12S-nematode, and 16S-helminth), 2,439,972 raw sequence reads were generated in total. Of these, 565,734 reads, 972,922 reads, and 901,316 reads were obtained from 12S-platyhelminth, 12S-nematode, and 16S-helminth, respectively. After merging the raw sequence reads that passed quality filtering, 254,393 reads were recovered for 12S-platyhelminth, 254,624 reads for 12S-nematode, and 404,123 reads for 16S-helminth. Among the three replicates of the no environment matrix mock community per genetic marker, the number of raw sequence reads and final filtered sequences obtained were similar, showing limited amounts of variability among PCR replicates. Comparing the proportion of raw sequence reads obtained between the 12S and 16S rRNA genes, more were obtained from the 12S rRNA gene. The number of raw sequence reads obtained and the percentage of target-specific sequences recovered for each type of mock helminth community are presented in Supplementary Table S1.

The percentage of target-specific sequences (platyhelminths and nematodes) recovered was calculated for each mock helminth community to determine the specificity and robustness of the 12S and 16S primers for helminth DNA metabarcoding. Using the 12S-platyhelminth primers, 100% of the filtered sequences obtained belonged to platyhelminths for each type of mock community. A similar result was observed with the 16S platyhelminth primers, where more than 94% of the sequences were platyhelminth specific (except the mock tissue community). For the 12S nematode primers, a low percentage of nematode-specific sequences were obtained. However, the 16S nematode primers performed significantly better than the 12S nematode primers, where more than 50% of the sequences were nematode specific (except the pond water mock community).

### Species recovery in mock helminth communities

**Figure 1:**
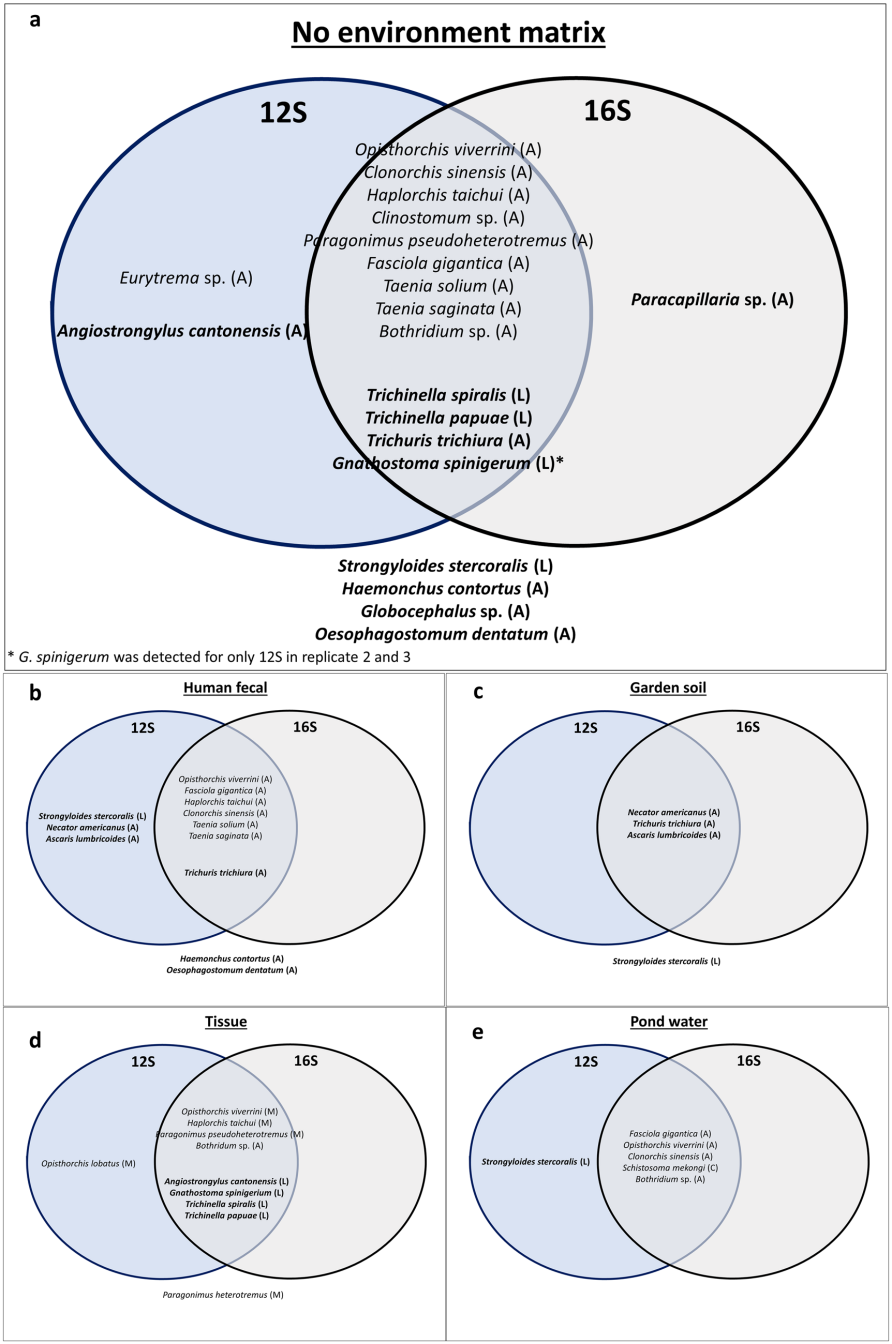
Venn diagram of helminth species recovered in the mock communities—(**a**) no environment matrix, (**b**) human fecal, (**c**) garden soil, (**d**) tissue, and (**e**) pond water after DNA metabarcoding using the mitochondrial 12S and 16S rRNA genes. Species recovered using both genetic markers are in the overlap region, while species not detected by either marker are outside of the Venn diagram. Nematodes are indicated in **bold** text, while platyhelminths are not in bold text.

With five types of mock helminth communities, the proportion of helminth species successfully recovered was obtained to determine the effectiveness and accuracy of the mitochondrial rRNA genes for helminth DNA metabarcoding. Figure 1a–e present the helminth species recovered in each type of community for the 12S and 16S rRNA genes. Firstly, comparing the genetic markers, the 12S rRNA gene recovered more helminth species for all the types of mock helminth communities. Secondly, both the 12S and 16S platyhelminth primers could recover a majority of the platyhelminth species in the mock communities, revealing the effectiveness of the primers for platyhelminth DNA metabarcoding. Contrarily, the 12S and 16S nematode primers recovered a lower percentage of nematode species. Thirdly, helminths of various life-cycle stages were successfully detected in the mock helminth communities regardless of the type of environment matrix.

#### No environment matrix

Twenty representative parasitic helminth species, comprising 10 platyhelminths and 10 nematodes, were selected to make up the no environment matrix mock helminth community. Three PCR replicates were performed for each genetic marker to determine whether PCR amplification bias could be present. The results revealed no significant difference between the replicates, with similar species recovered. The only exception was the larva of *Gnathostoma spinigerum,* where it was not recovered for the 16S rRNA gene in replicates 2 and 3, whereas *G. spinigerum* was recovered in replicate 1. As presented in Fig. 1a, 16 species were able to be successfully recovered with either of the genetic markers, and 13 species were recovered by both markers; out of the 20 parasitic helminth species present in this mock community.

Among platyhelminths, all the representative species were recovered with either genetic marker, with nine species recovered by both markers. The effectiveness of the 12S and 16S platyhelminth primers was revealed, with all 10 species successfully detected and accurately identified after phylogenetic analysis. On the other hand, the number of species recovered for nematodes was lesser than platyhelminths. Of the 10 nematode species present, six species were successfully recovered. With both genetic markers, four species were recovered: *Trichinella spiralis*, *Trichinella papuae*, *Trichuris trichiura*, and *Gnathostoma spinigerum*. *Angiostrongylus cantonesis* was detected with the 12S rRNA gene, while *Paracapillaria* sp. was detected with the 16S rRNA gene. However, *Strongyloides stercoralis*, *Haemonchus contortus*, *Oesophagostomum dentatum*, and *Globocephalus* sp. were not recovered by either genetic marker. The results suggest the lower sensitivity of the nematode primers compared with the platyhelminth primers and agree with the low percentage of nematode-specific sequences obtained after analysis (Supplementary Table S1).

#### Human fecal material

The human fecal mock helminth community is comprised of 12 parasitic helminth species, with six species each for platyhelminths and nematodes. Similarly, the results revealed that all six platyhelminth species were successfully recovered with both the 12S and 16S rRNA genes (Fig. 1b). The effectiveness of the 12S and 16S platyhelminth primers was also substantiated by having no difference in the percentage of platyhelminth species recovered between the no environment matrix mock helminth community despite spiking with fecal environment matrix. For nematodes, four species were successfully recovered among the two genetic markers, with *H. contortus* and *O. dentatum* not recovered for either. Additionally, the higher sensitivity of the 12S nematode primers compared to the 16S nematode primers was revealed, with *T. trichiura*, *Ascaris lumbricoides*, *S. stercoralis*, and *Necator americanus* being recovered using the 12S rRNA gene. In contrast, only *T. trichuira* was recovered using the 16S rRNA gene.

#### Garden soil

The garden soil mock helminth community consisted of soil-transmitted helminths of medical importance to humans, with four nematode species represented. As shown in Fig. 1c, adults of *T. trichuria*, *A. lumbricoides*, and *N. americanus* were successfully recovered with the 12S and 16S rRNA genes, while the larva of *S. stercoralis* was not able to be recovered. Contrarily, *S. stercoralis* larva was retrieved in the human fecal mock community using the 12S rRNA gene, thus possibly indicating that the type of environment matrix and differences in copy numbers between life-cycle stages affect helminth species detection.

#### Tissue

Helminth immatures make up a majority of species in the mock tissue community, with the metacercaria stage for platyhelminths and the larva stage for nematodes as they are frequently found in animal tissue samples. A total of six platyhelminths and four nematode species were represented in the mock tissue community. Five of the six platyhelminth species were successfully recovered, with *Opisthorchis viverrini*, *Opisthorchis lobatus*, *Haplorchis taichui*, *Paragonimus pseudoheterotremus*, and *Bothridium* sp. present (Fig. 1d). However, *Paragonimus heterotremus* was not detected with either genetic marker. For nematodes, all the species were successfully recovered with both genetic markers. The results indicate the ability of the primers to detect helminths of various life-cycle stage.

#### Pond water

The pond water mock community comprised six helminth species, with five platyhelminths and *S. stercoralis* as the only representative for nematodes. All six helminth species were recovered with either genetic marker, with all five platyhelminth species recovered with both the 12S and 16S rRNA genes (Fig. 1e). Additionally, the cercaria of *Schistosoma mekongi* was recovered, supporting the high sensitivity of the 12S and 16S primers for platyhelminths. Like the human fecal mock helminth community, *S. stercoralis* was only recovered with the 12S rRNA gene.

## Discussion

By measuring the species recovered in mock helminth communities with known species in five types of environmental matrices, we revealed the potential use of the mitochondrial 12S and 16S rRNA genes for DNA metabarcoding of parasitic helminths encompassing nematodes, trematodes, and cestodes. Firstly, compared to the 16S rRNA gene, the power of the mitochondrial 12S rRNA gene for helminth DNA metabarcoding was demonstrated. Secondly, the effectiveness of the platyhelminth primers was evidenced through their high sensitivity and specificity, with majority of the species recovered in all mock helminth community types.

First, our results demonstrate the robustness and sensitivity of the mitochondrial 12S rRNA gene primers to detect both platyhelminths and nematodes compared to the 16S rRNA gene. All the representative species that could be recovered were detected by the 12S rRNA gene, except for *Paracapillaria* sp., which was only detected with the 16S rRNA gene.

The robustness of the 12S primers is advantageous for eDNA metabarcoding, especially when species are detected directly from the environment. Differences in species composition are a factor in eDNA metabarcoding studies, where the species present in the majority often get selectively amplified^5,10,33^. Primers should ideally be highly sensitive to detect the taxa of interest, as helminths or eDNA from various species are usually not present in equal quantities in the environment. Moreover, helminths of various life-cycle stages can be found in the environment. Thus, with a robust and sensitive primer, some species or helminth immatures present in lower quantities or lower copy numbers have a chance of being detected. Here, in our artificially spiked mock helminth communities, we showed that our 12S primers could recover most platyhelminth and nematode species of various life-cycle stages compared to 16S.

The power of the mitochondrial 12S rRNA gene can be evidenced through its popularity as a genetic marker for DNA metabarcoding of fishes, marine mammals, and mammals^34–37^. Factors supplementing the 12S rRNA gene’s suitability for DNA metabarcoding include having universal primers targeting a broad species range within the taxa of interest, possessing high sensitivity, and containing sufficient information for species identification^38,39^. To date, the mitochondrial 12S rRNA gene has only been utilized for DNA metabarcoding of nematodes in museum preserved vertebrate specimens^40^. In this study, with our highly sensitive 12S primers targeting a broad species range for nematodes and platyhelminths, we demonstrate its potential as a robust genetic marker for helminth DNA metabarcoding.

Second, compared to nematodes, a higher proportion of platyhelminths was recovered for the mitochondrial 12S and 16S rRNA genes. Among all the mock helminth communities with both genetic markers, only the metacercaria of *P. heterotremus* was not successfully recovered in the mock tissue helminth community. The results contrast with nematodes, where more species were not recovered. Additionally, the 12S and 16S platyhelminth primers recovered a single cercaria of *S. mekongi* present in the pond water mock helminth community. Our results support the recent findings by Chan et al. (2022), where the high sensitivity of the mitochondrial 12S and 16S primers was revealed by successfully amplifying all representative trematode specimens of various life-cycle stages for DNA barcoding^30^. As research on platyhelminth DNA metabarcoding remains relatively unexplored, apart from the study by Douchet et al. (2021), having a highly sensitive primer for platyhelminth detection can be helpful^31^.

Aside from the high sensitivity of the 12S and 16S platyhelminth primers, another measure of effective DNA metabarcoding primers lies in their specificity. Based on our sequences obtained from the platyhelminth primers, more than 94% were platyhelminth specific (Supplementary Table S1). Although nematode-specific 18S primers have been developed, studies have revealed that most recovered sequences are non-nematode-specific^28,33^. Having taxa-specific primers are valuable, especially for eDNA metabarcoding, where primers should efficiently amplify target taxa and not amplify non-target DNA present in the environment^33,41^.

Taken together, the high sensitivity and specificity of the mitochondrial rRNA genes and primers used in this study substantiate its effectiveness and efficiency for platyhelminth DNA metabarcoding. Moreover, with scarcely any studies on platyhelminth DNA metabarcoding available to date, we offer a promising avenue for future platyhelminth DNA metabarcoding studies through applying the mitochondrial rRNA genes and primers in this study.

Finally, apart from having high sensitivity and specificity, the mitochondrial rRNA genes possess additional advantages contributing to their potential for helminth DNA metabarcoding. Species-level taxonomic resolution of the mitochondrial rRNA genes is more robust than the nuclear 18S rRNA gene. Using the nuclear 18S rRNA gene for species identification (especially closely related species) could be challenging due to its low sequence variability. In an evaluation of three 18S primers for nematodes, Ahmed et al. (2020) showed that some taxa present in mock communities could only be taxonomically assigned to the family or genus level^28^. Also, in an assessment of ten genetic markers commonly used for helminth molecular-based studies, the nuclear 18S rRNA gene had the lowest sequence variation across taxonomic categories. In contrast, the mitochondrial rRNA genes had sufficient sequence variation to differentiate between closely related species for nematodes, trematodes, and cestodes^42,43^. As DNA metabarcoding requires short diagnostic sequences (≤ 300 base pairs) to accommodate for Illumina sequencing and degraded eDNA present in environmental samples, the high sequence conservation of the 18S rRNA gene can limit its use as there might not be sufficient informative sites for accurate species identification^24,44^. Thus, the mitochondrial rRNA genes possessing sufficient sequence variation to assign taxa to the species level further augment their potential for helminth DNA metabarcoding.

The mitochondrial 12S and 16S rRNA gene primers targeted a broad range of parasitic nematodes, trematodes, and cestodes. We showed that parasitic nematodes belonging to the four clades (clades I, III, IV, and V), trematodes belonging to three orders (Plagiorchiida, Echinostomida, and Strigeida), and cestodes in two orders (Cyclophylllidea and Diphyllobothridea) were able to be successfully detected. Our results are congruent with Chan et al. (2020, 2022), where the same parasitic nematodes and trematodes were able to be identified via Sanger sequencing using the mitochondrial rRNA gene primers^29,30^. Through targeting a broad range of helminth species added in the mock communities, we validated that these primers can be applicable for various types of helminth DNA metabarcoding studies targeting different hosts and environment types. The species included in the mock communities can be found not only in humans, but also in intermediate and definitive hosts such as snails, fishes, crustaceans, rodents, pigs, and ruminants.

Thus, the validation of the mitochondrial rRNA gene primers through mock helminth communities supports their universality for broad helminth species, host types, and host habitats, enhancing their potential as DNA metabarcoding markers.

We also provide a highly sensitive, accurate, and specific assay for the concurrent detection of the three main groups of parasitic helminths (nematodes, trematodes, and cestodes). Helminth DNA metabarcoding studies that have been conducted thus far usually focus on each helminth group. Here, three groups of parasitic helminths can be targeted by utilizing three and four pairs of primers for the 16S and 12S rRNA genes, respectively. Although we targeted nematodes, trematodes, and cestodes for the 16S rRNA gene in a single Illumina sequencing run, the sensitivity of the 16S assay can potentially be improved if nematodes and platyhelminths were targeted separately. Nonetheless, our study provides a valuable resource demonstrating the feasibility of DNA metabarcoding for the main groups of parasitic helminths.

Taking these additional advantages into consideration – having a robust taxonomic level resolution, primers targeting broad species range within helminths, and an assay to simultaneously detect both nematodes and platyhelminths, the mitochondrial 12S and 16S rRNA genes has the edge over other genetic markers frequently utilized for parasitic helminth DNA metabarcoding.

### Limitations and recommendations for helminth DNA metabarcoding

Despite the promising potential use of the mitochondrial rRNA genes for helminth DNA metabarcoding, we observed some drawbacks that could be further improved on. Firstly, not all species were successfully detected in the mock helminth communities. The unsuccessful species recovery was prominent for nematodes compared to platyhelminths, especially with an increased number of species present in the mock helminth community. Moreover, the same species not detected via Illumina sequencing were able to be successfully amplified when single-species PCR was performed. A similar observation was also seen by Porazinska et al. (2009) using the nuclear rRNA genes, where not all species were accounted for in the mock nematode community^8^. A possible factor contributing to unsuccessful species recovery lies in the DNA extraction step, where if a complex mixture of specimens is present, reduced efficiency in obtaining maximum DNA yield for each specimen can result^14,33,45,46^. This study used only one replicate per mock helminth community and did not account for DNA extraction bias.

Similar to the above, helminth immatures such as the metacercaria of *P. heterotremus* were not detected and the larva of *S. stercoralis* could only be detected in some mock helminth communities. Therefore, we suggest that DNA extraction procedures should be optimized, especially when helminths of various life-cycle stages are present. For example, tissue homogenization should be thoroughly done with a tissue lyser via bead-beating, particularly for small specimens, and to aid in mechanically disrupting the cuticle of nematodes or eggshells. The thick wall of trematode metacercaria can also be broken down with the addition of enzymes during DNA extraction^32,47,48^. Also, DNA extraction is commonly performed using commercial kits, with varied kit performance depending on the sample type. Waeyenberge et al. (2019) evaluated 15 DNA extraction procedures in nematode mixtures and found varying degrees of DNA yield^25^. Additionally, replicate sampling can be performed on a particular community to detect as many species as possible^49,50^.

Lastly, as the mitochondrial rRNA genes are not as widely used as the nuclear 18S rRNA gene or the mitochondrial *COI* gene for helminths, the small number of sequences available in reference databases restricts their ability for taxonomic assignment. Without a comprehensive reference sequence library, the chances of sequences being assigned to the species level decrease. Although different sequences can be delimited based on the degree of sequence variation and phylogenetic analysis, the species status of the sequence will remain ‘unknown’ without any taxonomic assignment. The limited number of reference sequences available for the mitochondrial rRNA genes can be circumvented by first generating reference sequences for individual species through Sanger sequencing^17,18,31^. Generating single sequences per species using the mitochondrial rRNA genes will also aid in the build-up of a comprehensive reference library for future users and accelerate the genetic markers’ popularity.

In conclusion, we revealed the potential use of the mitochondrial 12S and 16S rRNA genes for DNA metabarcoding of parasitic nematodes, trematodes, and cestodes using mock helminth communities with artificially spiked environment matrices. The mitochondrial rRNA genes as alternative genetic markers for helminth DNA metabarcoding may be beneficial and contribute to future applications such as non-invasive sampling of parasitic helminths and helminth biodiversity assessments in the environment. Future research perspectives include *natural* validation of the DNA metabarcoding approach and expanding the reference library of the mitochondrial rRNA genes for helminths.

## Methods

### Sample preparation of mock communities and DNA extraction

Mock helminth communities were prepared using the archived specimens at the Department of Helminthology, Faculty of Tropical Medicine, Mahidol University, Thailand. Ethical clearance and experimental protocols were approved by the Animal Care and Use Committee, Faculty of Tropical Medicine, Mahidol University, Bangkok (No. FTM-ACUC 016/2021E). Helminths were identified to the species level based on morphological characters and preserved in 70% ethanol at − 20 °C. For the mock communities, helminths that had available partial 12S and 16S rRNA gene sequences from previous studies were selected^29,30^. Five types of mock helminth communities were simulated through spiking of helminth communities with environmental samples. They comprise–no environment matrix, human fecal material, garden soil, tissue, and pond water, mimicking examples of environmental samples commonly used to detect parasitic helminths. Three PCR replicates of the no environment matrix mock community were performed, with each replicate comprising 20 helminth species. Table 1 shows the species present in each type of mock community.

**Table 1:** Helminth taxa and stages included in each type of mock community.

Mock community	Platyhelminth	Nematode
No environment matrix	*Opisthorchis viverrini* (A)	*Trichinella spiralis* (L)
	*Clonorchis sinensis* (A)	*Trichinella papuae* (L)
	*Haplorchis taichui* (A)	*Trichuris trichiura* (A)
	*Paragonimus pseudoheterotremus* (A)	*Paracapillaria* sp. (A)
	*Fasciola gigantica* (A)	*Gnathostoma spinigerum* (L)
	*Eurytrema* sp. (A)	*Strongyloides stercoralis* (L)
	*Clinostomum* sp. (A)	*Haemonchus contortus* (A)
	*Taenia solium* (A)	*Angiostrongylus cantonensis* (A)
	*Taenia saginata* (A)	*Globocephalus* sp. (A)
	*Bothridium* sp. (A)	*Oesophagostomum dentatum* (A)
Human fecal	*Opisthorchis viverrini* (A)	*Trichuris trichiura* (A)
	*Clonorchis sinensis* (A)	*Ascaris lumbricoides* (A)
	*Haplorchis taichui* (A)	*Strongyloides stercoralis* (L)
	*Fasciola gigantica* (A)	*Haemonchus contortus* (A)
	*Taenia solium* (A)	*Necator americanus* (A)
	*Taenia saginata* (A)	*Oesophagostomum dentatum* (A)
Garden soil		*Trichuris trichiura* (A)
		*Ascaris lumbricoides* (A)
		*Strongyloides stercoralis* (L)
		*Necator americanus* (A)
Tissue	*Opisthorchis viverrini* (M)	*Trichinella spiralis* (L)
	*Opisthorchis lobatus* (M)	*Trichinella papuae* (L)
	*Haplorchis taichui* (M)	*Gnathostoma spinigerum* (L)
	*Paragonimus heterotremus* (M)	*Angiostrongylus cantonensis* (L)
	*Paragonimus pseudoheterotremus* (M)	
	*Bothridium* sp. (A)	
Pond water	*Opisthorchis viverrini* (A)	*Strongyloides stercoralis* (L)
	*Clonorchis sinensis* (A)	
	*Fasciola gigantica* (A)	
	*Schistosoma mekongi* (C)	
	*Bothridium* sp. (A)	

The stages of each specimen are in parentheses, with adult = A, larva = L, metacercaria = M, and cercaria = C.

Adult helminths were individually separated and washed thoroughly with sterile distilled water. A 1 mm piece of the tissue was removed for nematodes, while a flat 1 mm^2^ piece of the tissue was removed for platyhelminths. Due to the small size of helminth immatures, the whole specimen (e.g., larva of nematodes, cercaria, and metacercaria of trematodes) was directly used in the mock communities.

Once the helminth specimens were prepared, they were spiked into each type of environment, followed by DNA extraction. One specimen per species was used for each mock community. Each mock community was homogenized with silica beads in lysis buffer using a TissueLyser LT (Qiagen, Hilden, Germany) before DNA extraction.

#### Fecal and soil environment

Human fecal material and garden soil were individually sieved using a 40 μm wire sieve mesh, and approximately 0.25 g of environmental samples were placed in a 1.7 ml microcentrifuge tube for DNA extraction with their respective helminth mock community. Total genomic DNA was then isolated from each mock community using the QIAmp® PowerFecal® DNA kit (Qiagen, Hilden, Germany), following the manufacturer’s recommendations.

#### Pond water environment

Representative helminths were added into 15 ml of pond water and filtered through an 11 μm Whatman filter paper (Sigma Aldrich, Darmstadt, Germany). After filtration, the tip of the filter paper was cut (approximately 1 cm in length) and placed in a 1.7 ml microcentrifuge tube for DNA extraction. Total genomic DNA was isolated using the DNeasy® Blood & Tissue kit (Qiagen, Hilden, Germany), following the manufacturer’s recommendations.

#### No environment matrix and tissue environment

The selected specimens were placed directly in a 1.7 ml microcentrifuge tube, and total genomic DNA was isolated using the DNeasy® Blood & Tissue kit (Qiagen, Hilden, Germany) following the manufacturer’s recommendations. The metacercaria and larva stages of representative trematodes and nematodes were used in the tissue environment to mimic the parasitic helminth immature stages that are commonly found in animal tissues. As helminths are usually isolated during animal tissue examination prior to molecular analyses, the mock tissue environment was assumed to be similar to the no environment matrix mock environment.

### PCR amplification and library preparation of mock communities

Three sets of PCR reactions were performed for each mock community (thereby named 12S-platyhelminth, 12S-nematode, and 16S-helminth). For the 12S rRNA gene, two PCR reactions with primers targeting each helminth group (12S-platyhelminth and 12S-nematode) were conducted per mock community, while for the 16S rRNA gene, one PCR reaction with primers targeting both platyhelminths and nematodes (16S-helminth) was performed. We used the 12S and 16S locus-specific primers from Chan et al. (2020, 2022)^29,30^ that targeted parasitic nematodes and trematodes. We designed new primers targeting the 12S rRNA gene for cestodes in this study. Briefly, the primers were designed using the complete 12S rRNA gene sequences of 35 selected cestode species from the NCBI database. PCR were performed on representative cestode specimens to test the newly designed primers for its sensitivity and specificity. Reference sequences for the 12S rRNA gene of the cestode specimens used in this study were also generated by Sanger sequencing (Macrogen, Seoul, South Korea). For DNA metabarcoding, all the 12S and 16S primers were tagged with the Illumina adaptor overhang sequence (Macrogen, Seoul, South Korea). As more than one primer pair was used in the PCR reaction, multiplex PCR optimization was performed before use. PCR amplification was performed in a final volume of 30 μl containing 15 μl of 2X i-Taq™ mastermix (iNtRON Biotechnology, Gyeonggi, South Korea), 5 μm to 10 μm of each primer, and 2 μl of DNA template. The PCR was conducted in a thermocycler (Bio-rad, California, USA) with the following profiles: 94 °C for 2 min of initial denaturation, followed by 94 °C for 30 s, 52 °C to 60 °C for 45 s, and 72 °C for 1 min, followed by a final extension step at 72 °C for 5 min. The PCR primers and annealing temperatures are listed in Table 2. Amplicons were checked and visualized on a 1.5% agarose gel and stained with GelRed® (Thomas Scientific, New Jersey, USA). The amplicons were purified using the Geneaid PCR purification kit (Geneaid Biotech Ltd., Taipei, Taiwan) before sending to a commercial company for Illumina sequencing (Macrogen, Seoul, South Korea).

**Table 2:** 12S and 16S rRNA gene primer sequences targeting each helminth group and their respective annealing temperatures.

Target gene	Target helminth group	Primer name	Primer sequence (5′–3′)	Amplicon size (bp)	Annealing temperature (°C)	References
12S	Platyhelminth	12S-trematode-F	TCGTCGGCAGCGTCAGATGTGTATAAGAGACAG GTGCCAGCADYYGCGGTTA	371	60	^30^
		12S-trematode-R	GTCTCGTGGGCTCGGAGATGTGTATAAGAGACAG AGCAGCAYATHGACCTG			
		*12S-cestode-F	TCGTCGGCAGCGTCAGATGTGTATAAGAGACAG GTGCCAGCATCYGCGGTTA	483		
		*12S-cestode-R	GTCTCGTGGGCTCGGAGATGTGTATAAGAGACAG GGTGACGGGCGGTGTGTAC			
	Nematode	12S-nematodeC1-F	TCGTCGGCAGCGTCAGATGTGTATAAGAGACAG GTGCCAGCTAYCGCGGTTA	460	52	^29^
		12S-nematodeC1-R	GTCTCGTGGGCTCGGAGATGTGTATAAGAGACAG GRTGACGGGCRATATGTG			
		12S-nematodeC345-F	TCGTCGGCAGCGTCAGATGTGTATAAGAGACAG GTWCCAGAATAATCGGMTA			
		12S-nematodeC345-R	GTCTCGTGGGCTCGGAGATGTGTATAAGAGACAG ATTGAYGGATGRTTTGTRC			
16S	Helminth	16S-platyhelminth-F	TCGTCGGCAGCGTCAGATGTGTATAAGAGACAG GTGYDAAGGTAGSATAAT	379	58	^30^
		16S-platyhelminth-R	GTCTCGTGGGCTCGGAGATGTGTATAAGAGACAG CCGGTYTYAACTCARCTCAT			
		16S-nematodeC1-F	TCGTCGGCAGCGTCAGATGTGTATAAGAGACAG ACGAGAAGACCCTRGRAAYT	240	58	^29^
		16S-nematodeC1-R	GTCTCGTGGGCTCGGAGATGTGTATAAGAGACAG GRTYTAAACTCAAATCACG			
		16S-nematodeC345-F	TCGTCGGCAGCGTCAGATGTGTATAAGAGACAG AAGATAAGTCTTYGGAARYT			
		16S-nematodeC345-R	GTCTCGTGGGCTCGGAGATGTGTATAAGAGACAG GAAYTAAACTAATATCAMG			

*Indicates newly designed primers in this study.

The Illumina overhang adaptor sequences are underlined.

Library preparation was performed using the 16S metagenomic sequencing library preparation protocol with the Herculase II Fusion DNA Polymerase Nextera XT V2 kit (Agilent, California, USA). The library was sequenced on an Illumina MiSeq platform.

### Analysis of NGS data

The sequences obtained after Illumina sequencing were demultiplexed using the onboard Illumina MiSeq platform. Raw sequence reads were dividived into separate FASTQ files for each indexed sample utilizing the bcl2fastq package (https://support.illumina.com/sequencing/sequencing_software/bcl2fastq-conversion-software.html). The quality of the raw sequence reads obtained was checked via FastQC through the command line^51^. The raw sequence reads were imported into Geneious Prime® 2022.0.2 (http://www.geneious.com), where the Illumina overhang adaptors, primer sequences, and low-quality sequences below Phred score of 20 were trimmed via BBDuk. The subsequent filtered raw sequence reads were merged and sequences generated. The resulting sequences were then filtered based on the target amplicon size per genetic marker for each helminth group (371 bp and 483 bp for 12S-platyhelminth, 460 bp for 12S-nematode, 240 bp (nematode) and 379 bp (platyhelminth) for 16S-helminth). After filtering based on the target amplicon size, contigs were generated and exported to FASTA format. The target sequences generated per sample were aligned in ClustalX^52^ together with the 12S and 16S rRNA gene sequences of representative helminth species obtained from the NCBI GenBank database. The sequences of the reference helminth species used were from the complete mitochondrial genomes and the partial 12S and 16S rRNA gene sequences obtained by Chan et al. (2020, 2022) previously^29,30^ (Supplementary Table S2). To check for matching sequences to the reference helminth species present in each mock community, the aligned sequences were checked using Bioedit^53^. Sequences obtained were allowed no more than 3% nucleotide difference between the reference sequence to be determined as a successful species match^43^. Additionally, phylogenetic analysis was performed in MEGA X^54^ to confirm the phylogentic placements of the successful matching sequences. The helminth species recovered per mock community were then presented using a Venn diagram.

### Ethics declarations

Ethical clearance was provided by the Animal Care and Use Committee, Faculty of Tropical Medicine, Mahidol University, Bangkok (No. FTM-ACUC 016/2021E).

## Supplementary Information

Supplementary Tables.

## Data Availability

All data generated or analysed during this study are included in this published article (and its Supplementary Information files).
