# Supplementary material for: Sensitive and accurate DNA metabarcoding of parasitic helminth mock communities using the mitochondrial rRNA genes
Source: Sci Rep. 2022 Jun 15;12:9947. doi: 10.1038/s41598-022-14176-z (PMC9200835; doi:10.1038/s41598-022-14176-z)
Supplement: Supplementary file 1 — Supplementary Tables. [file 41598_2022_14176_MOESM1_ESM.pdf]

Sensitive and accurate DNA metabarcoding of parasitic helminth mock communities using the mitochondrial rRNA genes

Abigail Hui En Chan[1], Naowarat Saralamba[2], Sompob Saralamba[3], Jiraporn Ruangsittichai[4], Kittipong Chaisiri[1], Yanin Limpanont[5], Vachirapong Charoennitiwat[1], Urusa Thaenkham[1*]

[1]Department of Helminthology, Faculty of Tropical Medicine, Mahidol University, Bangkok, Thailand

[2]Department of Molecular Tropical Medicine and Genetics, Mahidol University, Bangkok, Thailand

[3]Mathematical and Economic Modelling (MAEMOD), Mahidol Oxford Tropical Medicine Research Unit, Faculty of Tropical Medicine, Mahidol University, Bangkok, Thailand

[4]Department of Medical Entomology, Faculty of Tropical Medicine, Mahidol University, Bangkok, Thailand

[5]Department of Social and Environmental Medicine, Faculty of Tropical Medicine, Mahidol University, Thailand

[*]Corresponding author

Supplementary Table S1. The number of raw sequence reads and final sequences obtained after NGS quality filtering, and percentage of target specific sequences obtained per mock helminth community

Genetic marker and helminth group	Mock helminth community	Number of total raw sequence reads	Number of merged raw sequence reads that passed quality filtering	Number of sequences obtained after amplicon size filtering	% of target specific sequences
12S-platyhelminth	Neat (replicate 1)	99,700	45,974	102	100.00
	Neat (replicate 2)	100,556	46,304	107	100.00
	Neat (replicate 3)	96,052	44,244	103	100.00
	Human fecal	90,790	40,719	38	100.00
	Tissue	83,166	34,012	25	100.00
	Pond water	95,470	43,140	33	100.00
12S-nematode	Neat (replicate 1)	123,882	43,309	165	4.24
	Neat (replicate 2)	123,438	44,275	144	6.25
	Neat (replicate 3)	128,556	44,471	152	5.92
	Human fecal	183,272	20,883	193	2.07
	Garden soil	109,614	26,489	160	10.62
	Tissue	121,906	46,564	40	82.5
	Pond water	182,254	28,633	138	0.72
16S-helminth	Neat (replicate 1)	131,396	41,720	302	96.36
			**21,324**	**64**	**67.19**
	Neat (replicate 2)	111,314	35,772	219	94.52
			**17,788**	**66**	**95.45**
	Neat (replicate 3)	118,034	36,708	258	95.74
			**19,729**	**56**	**94.64**
	Human fecal	139,288	47,012	146	99.31
			**7,938**	**49**	**51.02**
	Garden soil	118,878	NA	NA	NA
			**52,259**	**75**	**100.00**
	Tissue	161,124	17,045	52	55.77
			**48,840**	**156**	**100.00**
	Pond water	121,282	57,767	146	100.00
			**221**	**15**	**0**

The values in **bold** text for 16S-helminth indicate the data obtained for nematodes while those not in bold text indicate the data obtained for platyhelminths.

Supplementary Table S2. Mitochondrial 12S and 16S rRNA gene sequences used in analysis

Helminth group	Species	NCBI ascension number
Platyhelminth	*Opisthorchis viverrini*	**JF739555**<br>MZ331639/ MZ331603<br>MZ331640/ MZ331604
	Opisthorchis lobatus	MZ331637/ MZ331601<br>MZ331638/ MZ331602
	Clonorchis sinensis	**MT607652**<br>**KY564177**<br>**FJ381664**<br>**JF729304**<br>**JF729303**<br>MZ331635/ MZ331595<br>MZ331636/ MZ331596
	Haplorchis taichui	**KF214770**<br>**MG972809**<br>MZ331646/ MZ331605
	Paragonimus pseudoheterotremus	MZ331669/ MZ345700<br>MZ331670/ MZ345701
	Paragonimus heterotremus	**KY952166**<br>**MH059809**<br>MZ331665/ MZ345698<br>MZ331666/ MZ345699
	Clinostomum complanatum<br>*Clinostomum* sp.	**KM923964**<br>MZ331680/ MZ331631<br>MZ331681/ MZ331632
	Fasciola gigantica	**KF543342**<br>**MH621335**<br>MZ331676/ MZ331627<br>MZ331677/ MZ331628
	Eurytrema pancreaticum<br>*Eurytrema* sp.	**KP241855**<br>MZ331671/ MZ345704<br>MZ331672/ MZ345705<br>MZ331673/ MZ331634
	Schistosoma mekongi	**AF217449**<br>MZ331682/ MZ331633<br>MZ331683
	Taenia solium	**AB086256**
	Taenia saginata	**AY684274**
	Bothridium pithonis	**MW602526**
Nematode	*Trichuris trichiura*	**GU385218**<br>**KT449826**<br>**MW448472**

		**MW448470**<br>**KY368774**<br>**KY368773**<br>**KY368772**<br>**KY368771**<br>**KY368770**<br>**KY368769**<br>**KY368768**<br>**KY368766**<br>**KY368765**<br>MT135051/ <u>MT151872</u><br>MT135052/ <u>MT151873</u>
	Trichinella spiralis	**GU386314**<br>**AF293969**<br>**KM357422**<br>MT135055/ <u>MT151876</u><br>MT135056/ <u>MT151877</u>
	Trichinella papuae	**KM357417**<br>MT135053/ <u>MT151874</u><br>MT135054/ <u>MT151875</u>
	Capillaria sp.	**MH665363**
	Ascaris lumbricoides	**HQ704900**<br>**JN801161**<br>**MT135063**<br>**MT135064**
	Gnathostoma spinigerum	**KP410547**<br>MT135071/ <u>MT151890</u><br>MT135072/ <u>MT151891</u>
	Strongyloides stercoralis	**LC050212**<br>**MT135075**<br>**MT135076**
	Haemonchus contortus	**EU346694**<br>MT135077/ <u>MT151894</u><br>MT135078/ <u>MT151895</u>
	Angiostrongylus cantonensis	**MK570629**<br>**GQ398121**<br>**KT186242**<br>**MK570632**<br>**MK570631**<br>**MK570630**<br>MT135082/ <u>MT151899</u><br>MT135083/ <u>MT151900</u>
	Globocephalus sp.	MT135092/ <u>MT151909</u><br>MT135093/ <u>MT151910</u>
	Oesophagostomum dentatum	**GQ888716**<br>MT135084/ <u>MT151901</u>

		MT135085/ MT151902
	Necator americanus	**AJ417719**
		MT135090/ MT151907
		MT135091/ MT151908

Ascension numbers in **bold** were obtained from complete mitochondrial genomes, while those in normal text are the partial 12S rRNA gene sequences and those underlined are the partial 16S rRNA gene sequences.
